# Anaphylaxis Imaging: Non-Invasive Measurement of Surface Body Temperature and Physical Activity in Small Animals

**DOI:** 10.1371/journal.pone.0150819

**Published:** 2016-03-10

**Authors:** Krisztina Manzano-Szalai, Isabella Pali-Schöll, Durga Krishnamurthy, Caroline Stremnitzer, Ingo Flaschberger, Erika Jensen-Jarolim

**Affiliations:** 1 Comparative Medicine, Messerli Research Institute of the University of Veterinary Medicine Vienna, Medical University of Vienna and University of Vienna, Vienna, Austria; 2 Institute of Pathophysiology and Allergy Research, Center of Pathophysiology, Infectiology and Immunology, Medical University of Vienna, Vienna, Austria; 3 Crossip Communications GmbH, Vienna, Austria; Research Center Borstel, GERMANY

## Abstract

In highly sensitized patients, the encounter with a specific allergen from food, insect stings or medications may rapidly induce systemic anaphylaxis with potentially lethal symptoms. Countless animal models of anaphylaxis, most often in BALB/c mice, were established to understand the pathophysiology and to prove the safety of different treatments. The most common symptoms during anaphylactic shock are drop of body temperature and reduced physical activity. To refine, improve and objectify the currently applied manual monitoring methods, we developed an imaging method for the automated, non-invasive measurement of the whole-body surface temperature and, at the same time, of the horizontal and vertical movement activity of small animals. We tested the anaphylaxis imaging in three *in vivo* allergy mouse models for i) milk allergy, ii) peanut allergy and iii) egg allergy. These proof-of-principle experiments suggest that the imaging technology represents a reliable non-invasive method for the objective monitoring of small animals during anaphylaxis over time. We propose that the method will be useful for monitoring diseases associated with both, changes in body temperature and in physical behaviour.

## Introduction

Anaphylaxis is a severe, life-threatening hypersensitivity reaction that can be caused by immunological (IgE-dependent or independent) or non-immunological mechanisms [1]. Around 0.05–2.0% of people experience anaphylaxis due to insect bites/stings, food, or medications [2] and advances in prevention and therapy are urgently needed [3]. Symptoms like itchy rash, throat swelling, or rapid drop of blood pressure occur on average between 5 and 30 minutes after contact with the allergen. Affected body sites are the skin (80–90%), respiratory (70%) or gastrointestinal (30–45%) tract, heart and vascular system (10–45%), or central nervous system (10–15%) [4, 5]. In the pathophysiology of anaphylaxis, mediators released from mast cells or basophils, such as histamine, different interleukins such as IL-4, leukotrienes, and prostaglandins play an important role [6–8].

Animal models are useful in studying the mechanisms of food-induced anaphylaxis [9–11]. Usually, mice are sensitized intraperitoneally (i.p.), subcutaneously (s.c.), or by the oral route with allergens, for instance food allergens from milk [12, 13], peanut [14, 15], codfish [16], or egg [17]. After the successful specific sensitization, mice are mostly challenged intravenously (i.v.) with the corresponding allergen to induce anaphylaxis, with typical symptoms like hypothermia, reduced physical activity, diarrhoea and/or other parameters. Often the so-called anaphylaxis score is used: 0 –no symptoms; 1 –scratching around nose and head; 2 –puffiness around eyes and mouth; 3 –wheezing, laboured respiration, cyanosis around mouth and tail; 4 –no activity after prodding, or tremor and convulsion [18]. The monitoring of the drop in core body temperature in anaphylaxis is usually done by measurements of the core body temperature using hand-held thermometers rectally before and after challenge [19, 20]. Also rearing of mice as readout for physical activity is counted manually, which makes results very operator-dependent.

Therefore, we undertook efforts to automate data acquisition during anaphylaxis studies for increasing the objectiveness of results. We developed an imaging method for the non-invasive, continuous measurement of surface body temperature simultaneously with physical activity, allowing the monitoring of 4 small animals at a time [21].

In this study, we present the detailed description of the imaging technology for temperature measurement in comparison with the established manual method. In parallel to surface body temperature monitoring the images are further processed by a software that calculates the horizontally moved distance following the centre of heat points in the images, and the variations of vertical mouse movements based on the frequency of changes of mouse cross sectional image areas. The proof of principle is demonstrated using data from three independent anaphylaxis mouse models, immunized either with milk allergen casein [12], peanut allergen Ara h 2 [15] or egg allergen ovalbumin [22].

## Materials and Methods

### Construction of the imaging cage

**Fig 1:**
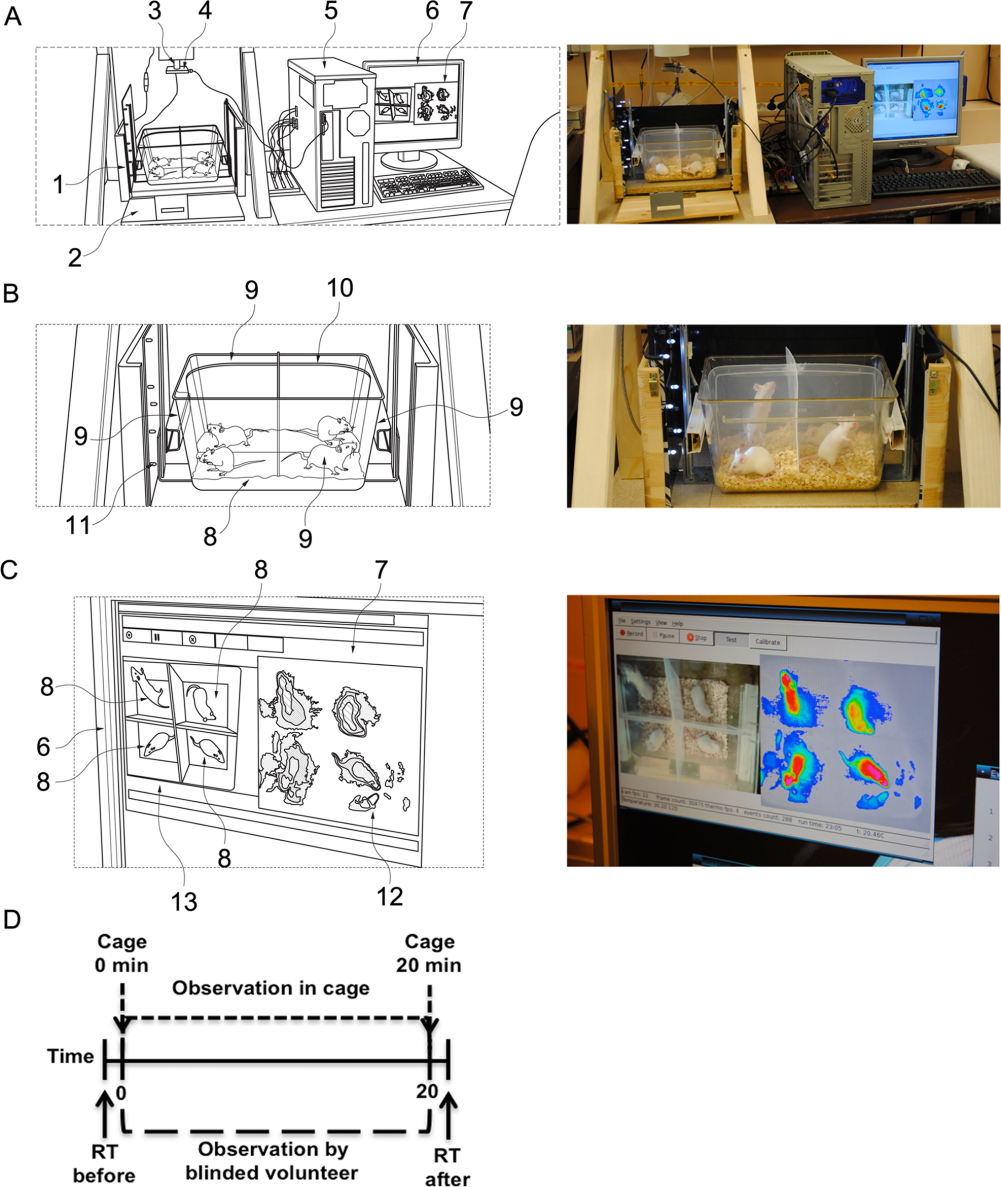
The schematic (left panels) and real set up (right panels) of the imaging cage. (A) The complete device is composed of a type II animal cage, a photo- and a thermo-camera, which are connected to a computer and software. (1) Outer box. (2) Outer sidewall. (3) Above the cage and height-adjustable, a heat camera or heat image camera (false colour infrared camera or infrared camera) is fixed with a reference heat electrode placed in the field of view for precise calibration against a reference temperature module. (4) Mounted true colour camera, which records live real image pictures during the experiment. (5) Data are recorded and processed, and translated by software to a personal computer for processing, identifying grey scales in video input from connected infrared camera 3. The software comprises a data processing part and (7) a graphical user interface, which is commonly displayed on (6) a screen. (B) Four small animals, in this case mice, can be placed and monitored at the same time in the cage. (8) Transparent enclosure adapted to house four mice. (9) The cage comprises four outer sidewalls and (10) two inner walls, as well as a bottom portion. (11) Vertical movements can be additionally recorded to the imaging data by photo sensors comprising transmitters and receivers. (C) The mice are individually monitored, where the thermo measurements and the live video from the cage appear at the same time on the monitor. (12) False colour screen output. Two pictures are recorded and may be visualized on screens. The software translates the grey image recordings from the infrared camera (Fig 1A-3) in real-time into a false colour screen output. (13) True colour image of the true colour camera is displayed in parallel. (D) Rectal temperature (RT) was measured manually before and 20 minutes after i.v. allergen challenge of hypersensitive or negative control mice. Immediately after the challenge, the same mice were placed into the imaging cage (0 min), and the cameras monitored surface body temperature and movement activity continuously for 20 minutes (20 min).

In principle, the imaging cage contains a transparent animal cage, as well as imaging cameras and infrared sensors connected to a computer (Fig 1A). The portable cage is embedded in an outer box (Fig 1B) containing photo sensors. Above the cage at adjustable height, a thermographic camera (false colour infrared camera) is placed with a reference heat probe located in the field of view for precise calibration of reference temperature. Next to the thermographic camera, an optical camera is mounted to record live pictures during the experiment. The data are recorded, processed and interpreted by a software, consisting of a data processing part and a graphical user interface (Fig 1C).

### Heat imaging for monitoring of surface body temperature and horizontally moved distances

**Fig 2:**
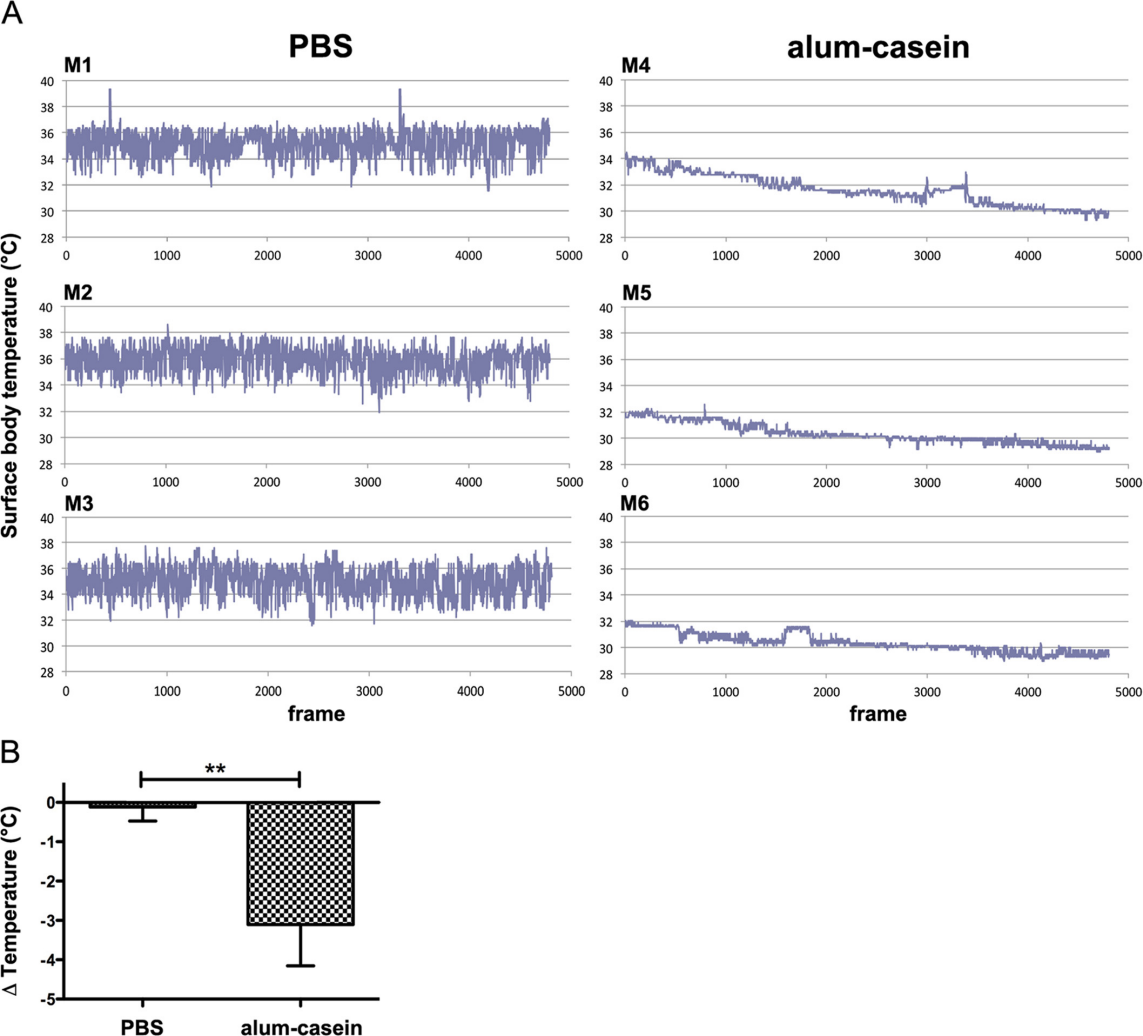
Surface body temperature imaging in the milk allergy model. Mice M1-M3 were sham-treated (PBS), M4-M6 were specifically sensitized with aluminium-absorbed milk allergen casein (alum-casein), all mice were i.v. challenged with casein and monitored in the imaging cage over 20 minutes, with an image frequency of 4 frames/sec. (A) Temperature curves of individual mice; y-axis represents temperature in °C, x-axis: number of frames during 20 min. (B) The mean results of drop of the body surface temperatures over time of each mouse group were compared and differed significantly (** p<0.01).

**Fig 3:**
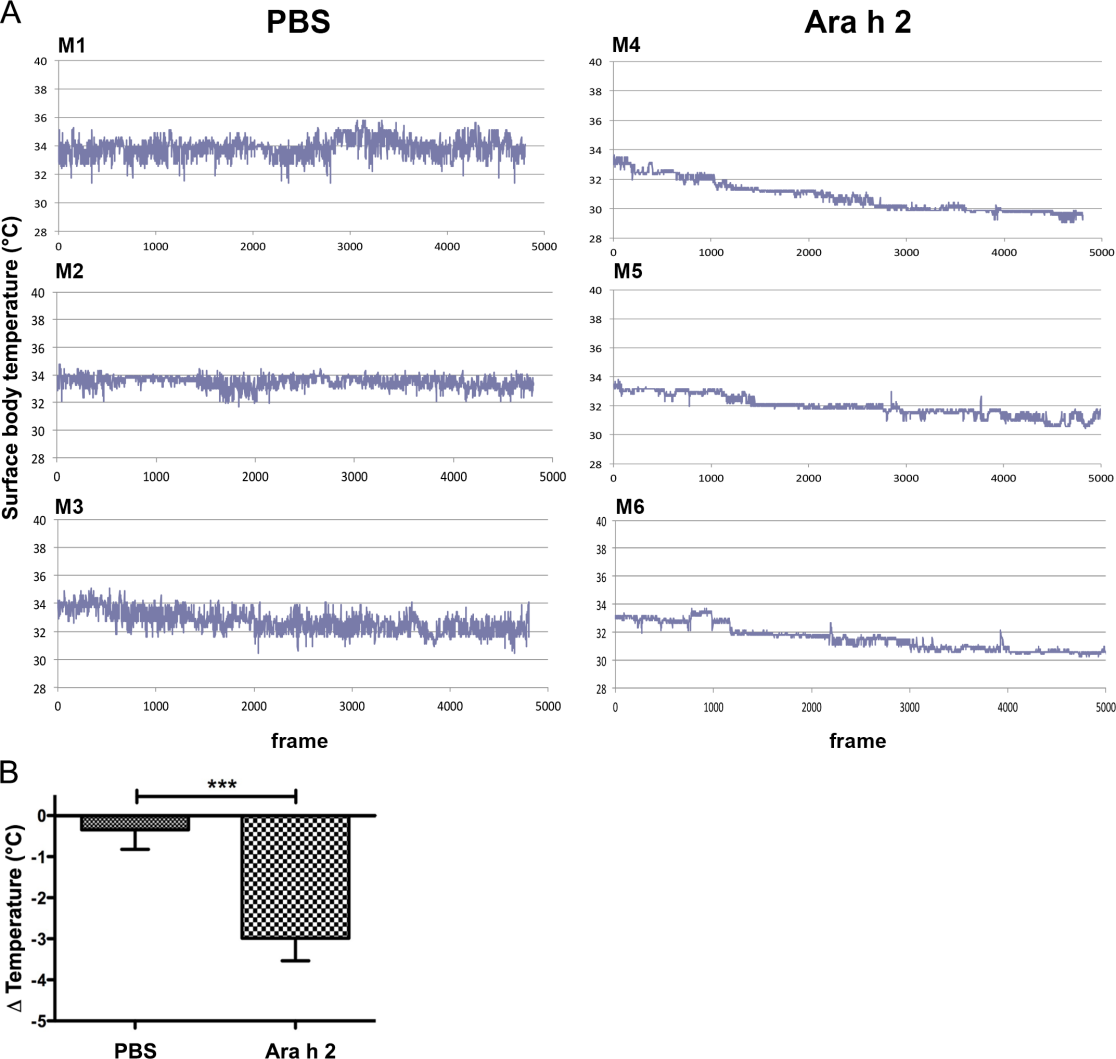
Monitoring of surface body temperature in the peanut allergy model. Mice M1-M3 were sham-treated (PBS), M4-M6 were specifically sensitized with peanut allergen Ara h 2 (Ara h 2), all mice were i.v. challenged with Ara h 2 and monitored in the imaging cage over 20 minutes, with an image frequency of 4 frames/sec. (A) Temperature curves of individual mice; y-axis represents temperature in °C, x-axis: number of frames during 20 min. (B) The mean drops of the body surface temperatures over time of each mouse group were compared and differed significantly (*** p<0.001).

**Fig 4:**
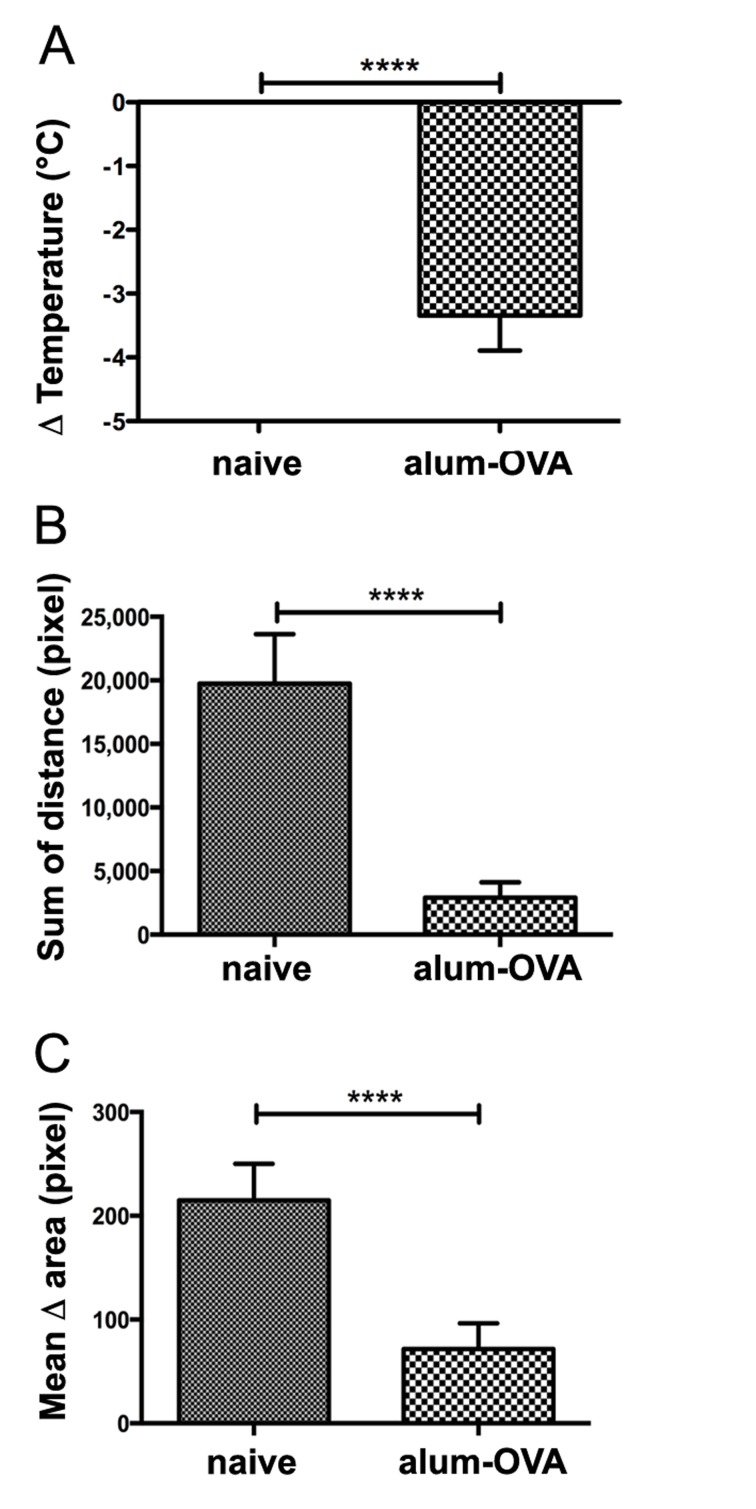
Monitoring body surface temperature, running and rearing activity in the egg allergy model. Mice remained untreated (naïve), or were specifically sensitized with egg allergen ovalbumin absorbed to aluminium hydroxide (alum-OVA), and all mice were i.v. challenged with OVA. (A) Mean surface body temperature drops recorded by imaging of naïve (0.49±0.26°C) versus alum-OVA sensitized mice (3.34±0.55°C) after allergen challenge in °C (**** p<0.0001). (B) The sum of horizontal move distances in pixels recorded by imaging over a period of 20 minutes, comparing naïve (19749.69±3887.62 pixel) versus alum-OVA sensitized mice (2909.54±1213.43 pixel) both being challenged with the specific allergen (**** p<0.0001). (C) The mean change (delta) of cross sectional image areas during 20 min significantly differ between the compared mouse groups (naïve: 214.9±35.19; alum-OVA sensitized: 71.62±24.72 pixel; **** p<0.0001).

**Fig 5:**
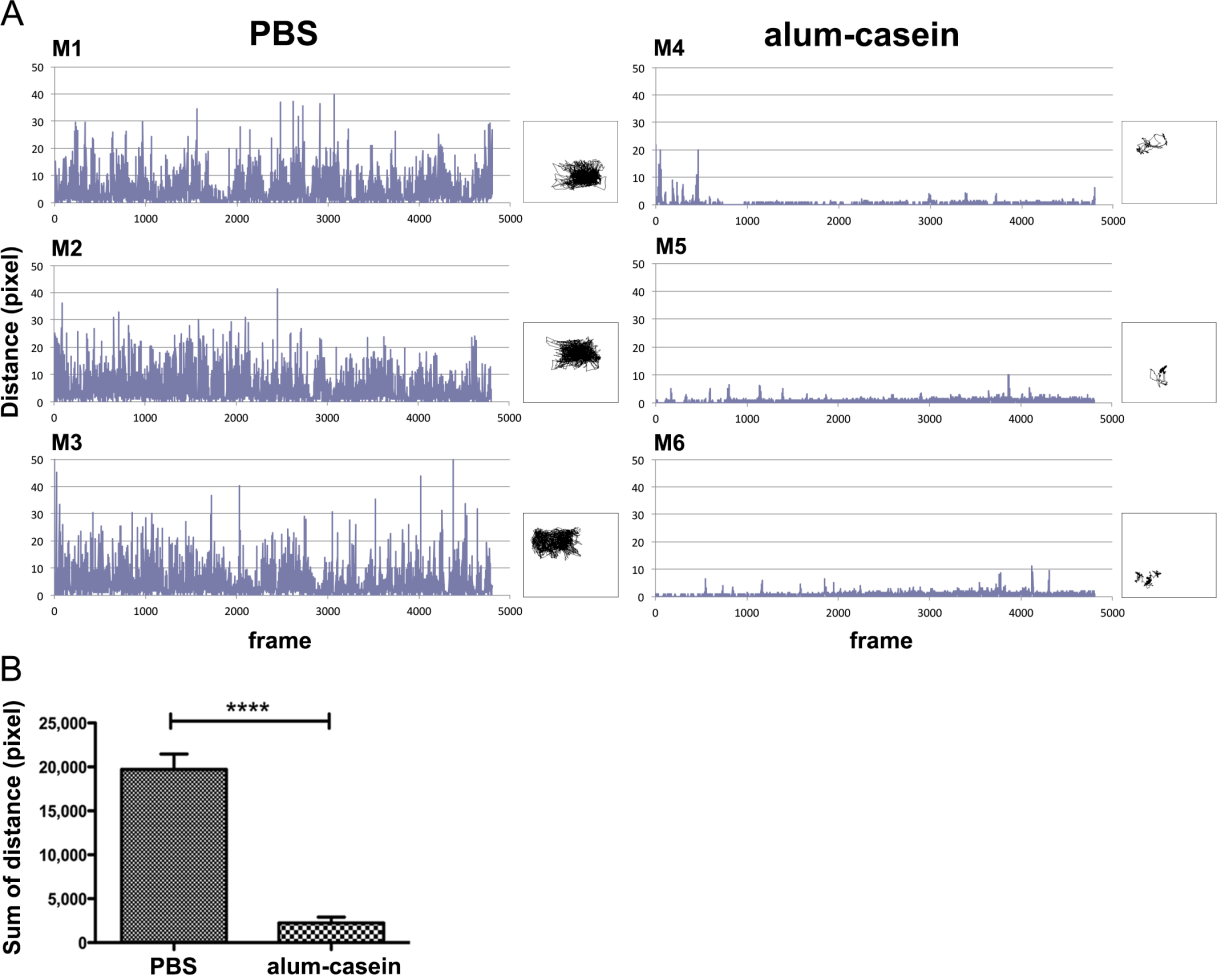
Horizontal physical activity of mice in the milk allergy model. Heat image frames of animals were captured (4 frames/second), and centre-of-heat-points and the distance among them calculated over a pre-set period of time. Y-axis: moved distance (pixel); x-axis: number of frames (images) captured during 20 min. Mice M1-M3 were sham-treated (PBS), M4-M6 were specifically sensitized against aluminium-absorbed milk allergen casein (alum-casein), all mice were i.v. challenged with casein. (A) Distance records of individual mice during 20 min; boxes: corresponding tracking curves of horizontal moves of each mouse. (B) Statistical comparison of the sum of moved distances between the PBS and alum-casein groups (mean+SD) showed a significant difference (****p<0.0001).

**Fig 6:**
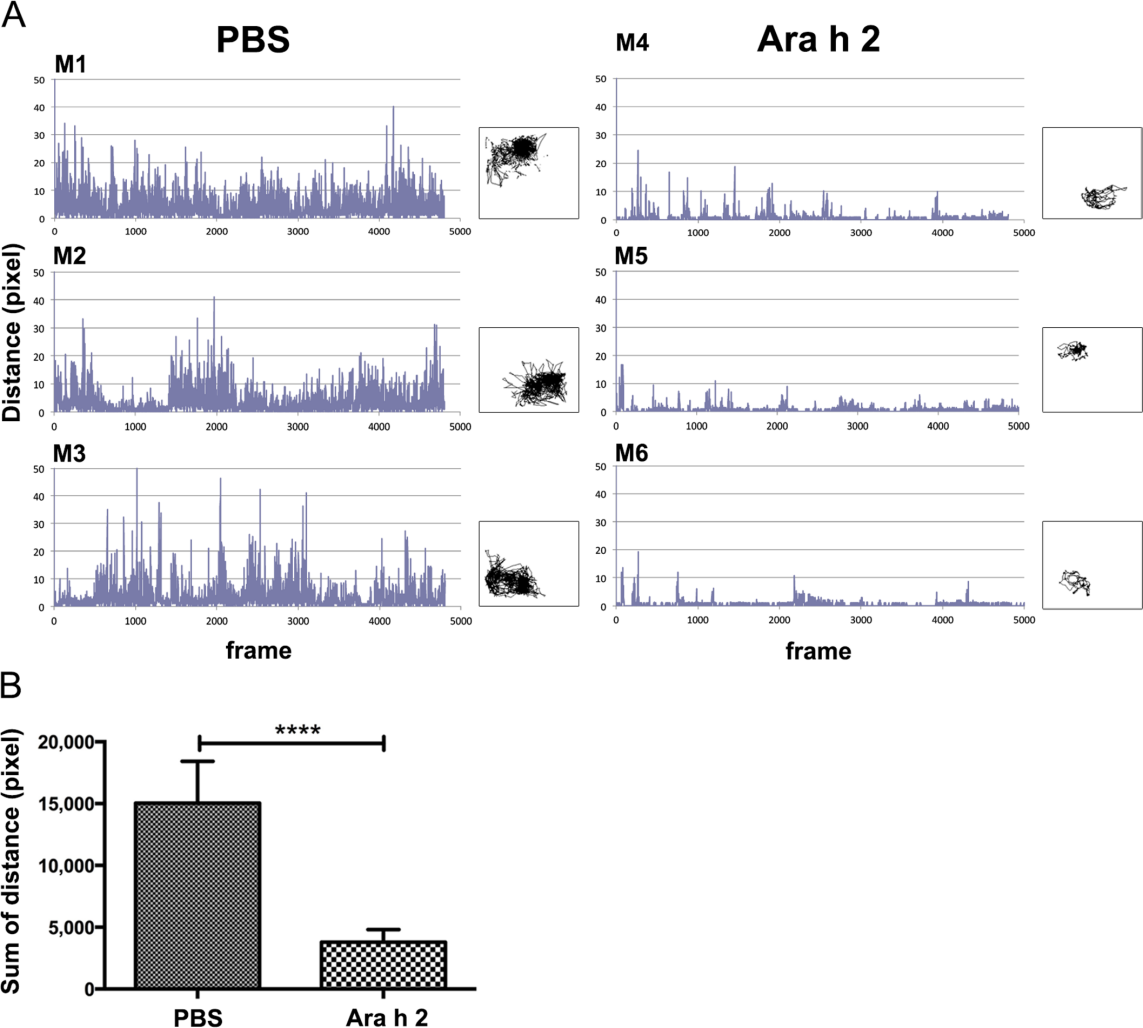
Horizontal physical activity of mice in the peanut allergy model. Heat image frames of animals are captured (4 frames/second), and centre-of-heat-points and the distance among them calculated over a pre-set period of time. Y-axis: moved distance (pixel); x-axis: number of frames (images) captured during the observation period of 20 min. Mice M1-M3 were sham-treated (PBS), M4-M6 were specifically sensitized against peanut allergen Ara h 2 (Ara h 2), and all mice were i.v. challenged with Ara h 2. (A) Distance records of individual mice during 20 min; boxes: corresponding tracking curves of horizontal moves of each mouse. (B) Statistical comparison of the sum of moved distances between the PBS and Ara h 2 groups (mean+SD) showed a significant difference (****p<0.0001).

**Fig 7:**
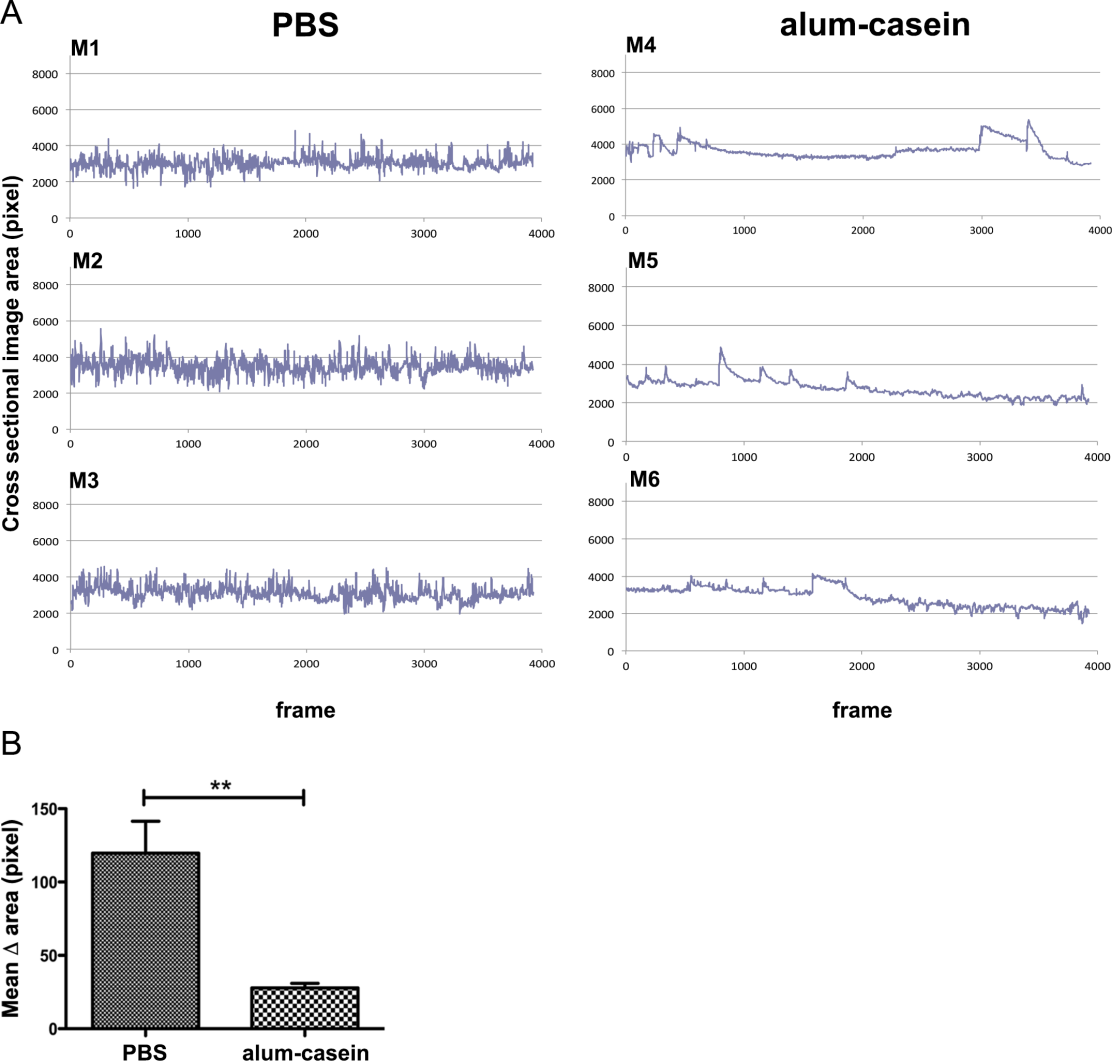
Monitoring of mouse vertical physical activity in the milk allergy model. (A) Readout of vertical physical activity of individual mice. Mice M1-M3 were sham-treated (PBS), M4-M6 were specifically sensitized with aluminium-absorbed milk allergen casein (alum-casein), and all mice were i.v. challenged with casein. The mouse cross sectional image area in pixels was recorded (y-axis). The number of signals over 16 min is given in the x-axis (4 frames/second). (B) Statistical analysis of the differences (delta) of mean values of cross sectional area changes in the PBS and alum-casein groups (** p = 0.0019).

The heat image camera (Fig 1A-3) is located above the cage and captures 4 heat images (i.e. frames) per second, here in total 4800 frames in 20 minutes, providing colour-coded 2-dimensional images. The system is adapted such that a temperature of around 40°C corresponds to a high, and around 25–30°C correspond to a low temperature. These temperatures generally correspond to high and low body temperatures of small rodent animals. During an experiment, two pictures are recorded and displayed on the connected screen (Fig 1A-6): the software (Fig 1A-7) translates the grey image recording from the infrared camera in real-time into a false colour screen output and records in parallel with the true colour camera (Fig 1A-4) real-time pictures of the cage. By means of the reference temperature module, the software translates grey scales into temperature values in the range from 25°C up to 40°C (as mentioned, the range of body temperature of small lab animals).

Also the centre-of-heat points for a sequence of images are calculated, giving a horizontal distance between the centre-of-heat points of sequential heat images. The resulting distances are used to determine the horizontal physical activity of the animals within a given time frame. A polyline, connecting the individual data points in a graph, represents the distance covered by the elapsed time.

### Monitoring of vertical movements by video imaging

A healthy mouse erects more than 10 times per minute within a cage, whereas a sick animal shows a reduced frequency of rearing. The number of erections can principally be counted by built-in photo sensors, but has been alternatively solved by the computer program using video images: Based on heat images the software calculates the average cross sectional areas of the mice that are characterized by a temperature within a defined threshold temperature range. With this computed area of shape, an anaphylactic mouse with a reduced frequency of area variations due to reduced physical activity can be recognized by the software and monitored over time.

### Animal experiments

Mice were obtained from the Institute for Laboratory Animal Science and Genetics (Medical University of Vienna) and treated according to European Community rules of animal care with the following permissions of the Austrian Ministry of Science: BWMF-66009/0123-II/10b/2008, BMWF-66.009/0172-II/3b/2011, and BMWF-66.009/0003-II/3b/2011.

After immunizations (see paragraphs below for more details), antigen challenges were induced by intravenous (i.v.) injection of the specific allergen into the tail vein. To measure the core body temperature of mice, rectal manual measurements were carried out right before challenge (first data point, before i.v. application of allergen) as well as 20 minutes after allergen challenge (second data point at end of observation period) (Fig 1D). Immediately after i.v. allergen challenge, mice were placed into the imaging cage for continuous calorimetric and movement monitoring over time (Fig 1D). Four mice were placed in the cage at the same time.

#### Experiment 1: Mouse model of anaphylaxis using milk allergen casein

Casein-specific hypersensitivity was induced in female BALB/c mice as described previously [12]. Briefly, 6–8 weeks old female mice (n = 3/group) were injected i.p. with 100 μg alum-adsorbed casein (Sigma, C6554), twice in two-weekly intervals (positive control group “alum-casein”), or sham-treated with PBS (negative control group “PBS”), and i.v. challenged with 100 μg casein in 100 μl 0.9% NaCl on day 113. Body surface temperature, core temperature as well as horizontal distances and vertical movement activity were monitored.

#### Experiment 2: Mouse model of anaphylaxis using peanut allergen Ara h 2

Allergy against the peanut allergen Ara h 2 was induced as previously published [15]. Briefly, female BALB/c mice (aged 8 weeks) were injected s.c. 4 times in bi-weekly intervals (n = 8/group) with 10 μg recombinant peanut allergen Ara h 2 in 100 μl PBS (“Ara h 2”), or sham-treated with 100 μl PBS (“PBS”), and challenged i.v. on day 70 with Ara h 2 (50 μl of 1 mg/ml in 0.9% NaCl). Body surface temperature, the horizontal distances covered, and vertical movement activity were monitored. The temperature results were already presented in [15], but are included here for convenient comparison of data.

#### Experiment 3: Mouse model of anaphylaxis using egg allergen ovalbumin

We investigated previously the safety of a therapeutic egg allergy vaccine based on Adeno-associated virus-like particles (AAVLPs) [22]. Female BALB/c mice (n = 5/group) were injected subcutaneously (s.c.) with alum-adsorbed ovalbumin (OVA) (positive control “alum-OVA”) three times in biweekly intervals, or were left untreated (“naïve”), and on day 41 challenged i.v. by 50μg OVA in 50μl 0.9% NaCl. Even though the temperature and horizontal movement results of the positive and negative groups were presented in [22], they are included here for direct comparison of the technical data. Data of physical activity were also collected by a blinded observer (Fig 1D), published in [22].

### Statistical analysis

Data analysis was done by GraphPad Prism 5 software (GraphPad, San Diego, CA, USA). The body temperature changes before vs. after i.v. challenge either gained by rectal temperature measurements or by the imaging method were compared by two-way Anova plus Bonferroni multiple correction. Multiplicity adjusted P-values <0.05 were considered statistically significant. All other analyses were performed using unpaired *t*-test, p values <0.05 were considered statistically significant.

## Results

### Imaging of body temperature drop during anaphylaxis

In the first experiment, BALB/c mice were sensitized with aluminium-absorbed casein, while a control group was sham-treated with PBS. Casein-specific IgG1 antibody levels, measured in ELISA, reached 41.5 ± 1.1 μg/ml mean titre, allergen-specific IgE 7.3 ± 0.31 μg/ml. The automated imaging cage method identified the head and eyes as the center of heat points of the body surface. When mice were challenged i.v. with the specific allergen, only the mice that were immunized with casein, but not the control group immediately reacted with a drop of surface body temperature within the 20 min observation period (Fig 2).

Mice from the PBS-group showed a constant body surface temperature upon i.v. casein challenge during the whole observation period, whereas in casein-allergic mice the body surface temperature declined significantly (Fig 2A and Table 1). Typical imaging videos during an anaphylaxis experiment are shown in a movie (S1 Movie.). Hence, the mean surface body temperature drop in the alum-casein group (3.1±1.05°C) was significantly higher than in the PBS group (0.11±0.36°C) (p<0.01) (Fig 2B). Due to the immediate onset of anaphylaxis upon i.v. injection, even a minimal delay in putting mice back into the cage may result in falsification of the data; the body surface temperature curves of mice M5 and M6 in this first experiment start at 32°C instead of 35°C. This should be improved by timely handling in further experiments.

**Table 1:** Comparison of body temperature measurements: rectally by hand-held thermometer (core body temperature) versus imaging cage (surface body temperature) before and after allergen challenge in 2 different allergy mouse models.

Immunization group	Core body temperature (°C)		p-value	Surface body temperature (°C)		p-value
	Before challenge	After challenge		0 min after challenge	20 min after challenge	
**Casein-immunization experiment**						
**PBS**	38.20±0.26	37.97±0.25	n.s.	35.63±0.65	35.52±0.29	n.s.
**alum-casein**	38.07±0.25	31.23±1.53	****	32.7±1.39	29.6±0.36	**
**OVA-immunization experiment**						
**Naive**	36.98±0.43	37.68±0.55	n.s.	32.35±0.64	32.85±0.49	n.s.
**alum-OVA**	36.16±0.69	30.58±1.03	****	31.46±0.68	28.12±0.36	****

Values represent means±SD of group. P-values for statistical analysis of means of groups before vs. after challenge are shown.

****P <0.0001

**P<0.01, n.s. = not statistically significant.

The results of the imaging method of time point 0 and 20 min were compared to results from manual temperature measurements before and after allergen challenge (Table 1A). Even though both methods reached statistical significance, the mean temperature drops were higher using the manual method (in the casein sensitive group: 6.83±1.51°C by manual method vs. 3.1±1.05°C by imaging). Hence, core body temperature measurements result in more pronounced temperature drops but punctual data, whereas the imaging method continuously follows temperature changes over time.

In the second experiment, we used the imaging cage to monitor anaphylactic reactions in a peanut-allergy mouse model. As previously described in [15], mice of the positive control group “Ara h 2” produced high levels of allergen-specific antibodies, and anaphylaxis could be induced when challenging with this peanut allergen.

The monitoring curves of three representative mice from each group are shown in Fig 3A. A statistically significantly higher drop of body surface temperature (Fig 3B) was recorded in Ara h 2-sensitized animals (2.99±0.33°C) within 20 minutes after putting them into the cage upon i.v. challenge with the specific allergen than in the group sham-treated with PBS (0.35±0.38°C), as reported previously [15].

In the third experiment, previously published data from the positive and negative control mouse groups of a therapeutic egg-allergy mouse model [22] were extracted. When the positive control group immunized with alum-OVA was challenged with OVA, a rapid drop of body surface temperature from mean 31.46±0.68 to 28.12±0.68°C was observed (Fig 4A) and ref. [22]). Similarly as in the milk allergy experiments, the delta of temperature was larger when measuring the body core temperature (5.58±0.84°C vs. 3.34±0.55°C) (Table 1).

### Imaging of horizontal movement activity during anaphylaxis

In addition to the body surface temperature of the animals, in all experiments running activity in terms of the distances moved horizontally by the mice were recorded following their centre of heat points in the videos. In the casein allergy model, individual movement data from naïve and allergic groups, both challenged with the allergen, are presented in Fig 5, where Fig 5A shows the horizontal movements of six individual mice over time as well as their individual tracks. In Fig 5B, the differences of distance were calculated for the two mouse groups. The data illustrate that upon the specific allergen challenge only the horizontal physical capacity of specifically sensitized mice was significantly impaired (Fig 5B).

Moreover, also in the peanut allergy model, the horizontal physical activity of individual mice was reduced after i.v. allergen challenge in the group immunized with Ara h 2, compared to the negative control group PBS (Fig 6). This result was confirmed by a significant difference in the sum of moved distances between these groups (Fig 6B).

Also in the egg allergy model, the distance moved by anaphylactic mice of the alum-OVA group was significantly reduced, while the naïve group showed steady physical activity (Fig 4B; single data published in [22].

### Imaging of rearing activity during anaphylaxis

The frequency of changes in cross sectional areas of each mouse was used to assess the vertical physical activity. Fig 7A illustrates in three individual mice that sham-treated mice “PBS”, when challenged with casein, during the whole observation time frequently changed the cross sectional body areas due to higher frequency of rearing. In contrast, the “alum-casein” sensitized mice upon challenge with the specific allergen showed less variations in the cross sectional areas as an indicator for reduced physical activity. There was a significant difference between the means of variation (delta) in cross sectional areas between the PBS and alum-casein group (Fig 7B).

**Fig 8:**
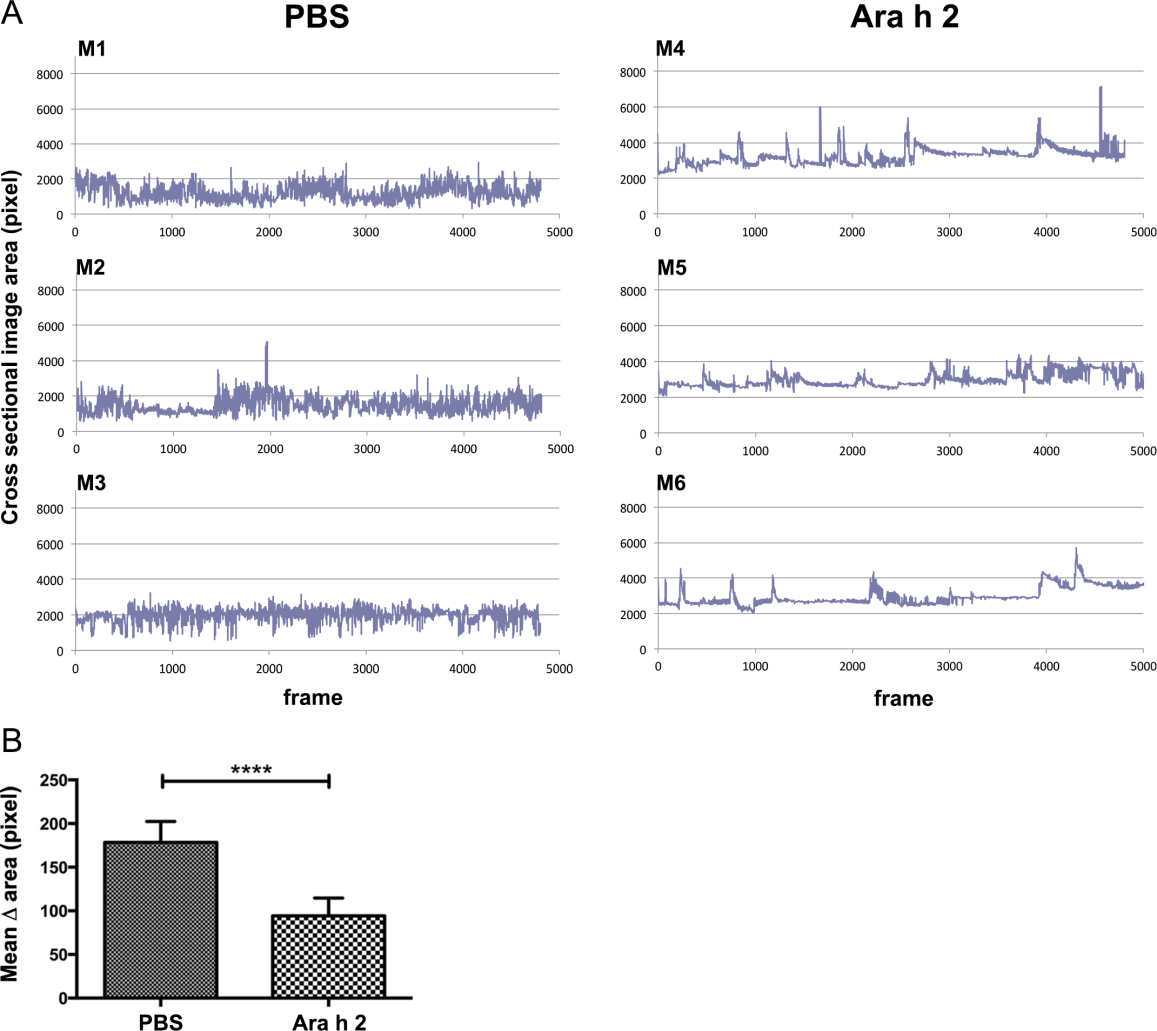
Monitoring of mouse rearing activity in the peanut allergy model. (A) Readout of vertical physical activity of individual mice. Mice M1-M3 were sham-treated (PBS), M4-M6 were specifically sensitized against peanut allergen Ara h 2 (Ara h 2), and all mice were i.v. challenged with Ara h 2. The mouse cross sectional image area in pixels was recorded (y-axis). The number of signals over 20 min is given in the x-axis (4 frames/second). The frequency of the cross sectional area changes and the area variations drastically decreased in the anaphylactic Ara h 2-sensitized group, while the PBS-group with high frequency and big variations of area changes remained stable during 20 minutes. (B) Statistical analysis of the difference of mean values of changes in the cross sectional area (delta) of the PBS and alum-casein groups (**** p<0.0001).

The same observation was made in the peanut allergen model. Ara h 2 challenges did not influence the frequency and variations of cross sectional area changes, i.e. rearing activities, of individual mice when they were sham-treated with PBS, but only when sensitized against Ara h 2 (Fig 8A). Comparing the mean values of cross sectional area changes (delta) of the PBS and Ara h 2 groups showed a statistically significant difference (Fig 8B).

The method of monitoring the frequency of changes in cross sectional areas to assess their vertical physical activity was also tested in the egg anaphylaxis mouse model. Only in the alum-OVA immunized group, but not in naïve mice, the i.v. OVA challenge resulted in significantly reduced frequency of the cross sectional area changes and the area variations, i.e. reduced rearing activity (Fig 4C).

## Discussion

In light of the increasing incidences and awareness of anaphylaxis [3], animal models are urgently needed to study its mechanisms, to improve its prevention and treatment. For these reasons, these models are also applied to study the safety profile of new vaccines. In mouse models of anaphylaxis, mice are first sensitized to a specific allergen and consecutively are challenged with the same allergen. Up to now, symptoms like hypothermia have been assessed by hand-held rectal measurements, and as readout for physical activity frequency of rearing of mice has been counted manually. Additionally, individual, blinded observers evaluate the anaphylaxis score in an attempt to objectify the collected data.

Pure video imaging of animals using optical cameras is a well-established method in behavioural studies, for instance to monitor stress-related behavioural changes [23]. Further, body temperature measurement in laboratory animals by thermo-cameras has been described [24], and even the 3-dimensional optical monitoring of body movements of zebra fish has been developed recently [25].

We have applied thermographic imaging for automated temperature evaluation during proof of concept experiments for allergen vaccines based on novel carrier systems [15, 22]. In contrast to the exclusive therapeutic aims of these previous studies, where temperature was only a minor aspect among many parameters, we present here the detailed and complete description of the imaging technology, which besides body surface temperature monitoring allows recording of horizontal and vertical movements of mice. Specifically in anaphylaxis these physiological parameters change significantly and can be evaluated by the thermographic imaging in a non-invasive and objective fashion. Here, a thermo-camera continuously measures the surface body temperature over a pre-set time of four individual mice at the same time (Figs 2A, 3A and 4A). The center of heat points of the animals, usually in the head, are used by the software for tracking their horizontal moves (Figs 4B, 5 and 6), whereas the frequency of changes in body shape (cross sectional area) turned out useful for monitoring rearing activities (Figs 4C, 7 and 8). Both are useful novel parameters in mouse anaphylaxis studies.

Body temperature changes go along with changes in physical activity in many diseases. The most significant changes in these parameters, however, occur during allergic anaphylaxis, when in an allergic individual an allergen reaches and crosslinks many IgE-sensitized allergy effector cells at the same time. The immediate release of vasodilatory mediators such as histamine may then lead to shock. As a counterreaction, a massive release of stress hormones leads to peripheral vasoconstriction resulting in a drop of the peripheral body temperature. In the US, the fatality rate due to anaphylactic reactions in general is 0.25% and 0.33% among hospitalizations or emergency department presentations, respectively [26]. Food is the most important trigger for anaphylaxis in infants to young adults, with an incidence rate of 0.14 in 100 persons/year at all ages [3]. Previously, we investigated food-induced anaphylaxis in several models with conventional handheld temperature measurement methods [11–13, 16, 27]. We were prompted to develop an improved combined method for the monitoring of symptoms during anaphylaxis for the following reasons:

- first, to have a non-invasive method to measure body temperature and physical behaviour which does not disturb animals or apply unnecessary distress by manipulation during the experiment;
- second, to create a truly objective observation method independent of the experimenter;
- third, to enable continuous data generation over an intended, prolonged period of time, also allowing longitudinal studies within one animal.

Milk, peanut and egg are important causes of allergy in children with anaphylactic risk [28]. In the milk [12] and egg anaphylaxis [22] mouse models, where we used casein or ovalbumin as allergens, respectively, we applied the imaging method in addition to manual recording of rectal temperature. Significant drops in body temperature (Figs 2B, 3B and 4A) could be recorded by both methods, with mean delta between 2.99 and 3.3°C per group by the imaging, and between 5.6 and 6.8°C by the manual method, comparable to data from independent experiments [27]. The basal levels of body temperature were lower in the cage measurements (Table 1) than by hand-held devices, and a smaller delta between baseline and anaphylactic temperature was observed in the imaging system, which reflects the difference between body core and surface temperature. Therefore, more studies are needed to explore whether the imaging cage will be useful for evaluating e.g. therapeutic approaches in anaphylaxis mouse models, although our proof of concept experiments support its usefulness. It is also recommended to record the baseline temperature of each mouse before the anaphylaxis experiment. Still, the surface body temperature data recorded by imaging parallel the manual core body temperature measurements, are objective and offer continuous data collection of simultaneously four animals. This also provides the opportunity to monitor animals from different groups at the same time (e.g. negative vs. positive control group) and to directly compare their pathophysiological symptoms as illustrated in our movie (S1 Movie). In contrast, the collection of data by hand-held instruments may vary from mouse to mouse, dependent on the experimenter, the time point of measurement, and the instrument used.

In parallel with the surface body temperature of the animals, the horizontal (Figs 4B, 5 and 6) and vertical (Figs 4C, 7 and 8) movement activity were recorded and automatically evaluated by the imaging software. So far, in mouse studies horizontal move distances have not been evaluated, but only manual counting of rearing, which may again be biased by the experimenter. In the three proof-of-concept experiments, both movement parameters changed significantly during anaphylaxis, the vertical rearing activity of mice represented by less frequent changes in their cross sectional image areas, as well as reduction of their horizontal move distances. Therefore, the imaging cage introduces a novel, objective method to precisely record not only surface body temperature, but also movements along both axes.

As many laboratories within different fields of science are interested in mouse studies, and as temperature is an important indicator of local or systemic inflammation, the method may be applicable in many physiological or behavioural problems connected with changes in body temperature and/or physical activity, where short- or long-term monitoring is necessary. The imaging cage represents a non-invasive method, which conforms to the “refinement” within the 3R-rules of animal experiments [29].

## Supporting Information

S1 Movie Video of typical real colour and heat images during an anaphylaxis imaging experiment. The visual monitoring of temperature changes of 4 mice in an imaging cage in a movie. Besides the heat camera pictures the real colour images are shown in the upper right corner. In the heat pictures, areas of highest temperature are shown in red, areas of lower temperatures in yellow, green and lowest in blue. Four mice, A, B, C, D, were simultaneously challenged with different antigens and monitored during 20 minutes. A, top left: mouse sensitized with alum-casein, challenged with PBS; B, top right: untreated, challenged with PBS; C, bottom left: sensitized with alum-casein, challenged with irrelevant antigen; D, bottom right: sensitized with alum-casein, and challenged with casein. Immediately after challenge with the specific antigen a decline in physical activity can be observed in mouse D only. This is followed with some delay by a drop of the surface body temperature, visualized by colour changes from red—yellow–green—blue during the observation period. Mouse D recovered from the anaphylaxis after 1 hour.
(MOV)

## Abbreviations

AAVLP: Adeno-associated virus-like particles
Alum: aluminium
ELISA: enzyme-linked immunosorbent assay
i.p.: intraperitoneal
i.v.: intravenous
min: minutes
OVA: ovalbumin
PBS: phosphate-buffered saline
RT: rectal temperature
s.c.: subcutaneous
SD: standard deviation
